# Associations between general practice characteristics with use of urgent referrals for suspected cancer and endoscopies: a cross-sectional ecological study

**DOI:** 10.1093/fampra/cmy118

**Published:** 2018-12-12

**Authors:** Silvia C Mendonca, Gary A Abel, Carolynn Gildea, Sean McPhail, Michael D Peake, Greg Rubin, Hardeep Singh, Willie Hamilton, Fiona M Walter, Martin O Roland, Georgios Lyratzopoulos

**Affiliations:** 1 The Health Improvement Institute (THIS), University of Cambridge, Cambridge, UK; 2 University of Exeter Medical School (Primary Care), Exeter, UK; 3 National Cancer Analysis and Registration Service (NCRAS), Public Health England, London, UK; 4 University of Leicester, Leicester, UK; 5 Institute of Health and Society, Newcastle University, Sir James Spence Institute, Newcastle upon Tyne, UK; 6 Houston VA Center for Innovations in Quality, Effectiveness and Safety, Michael E. DeBakey VA Medical Center and Baylor College of Medicine, Houston, TX, USA; 7 The Primary Care Unit, Department of Public Health and Primary Care, University of Cambridge, Cambridge, UK; 8 Cambridge Centre for Health Services Research, Department of Public Health and Primary Care, University of Cambridge, Cambridge, UK; 9 Epidemiology of Cancer Healthcare and Outcomes (ECHO) Research Group, Department of Behavioural Science and Health, University College London, London, UK

**Keywords:** Colonoscopy, gastroscopy, general Practice, neoplasms, outcome assessment (health care), referral and consultation

## Abstract

**Background:**

Large variation in measures of diagnostic activity has been described previously between English general practices, but related predictors remain understudied.

**Objective:**

To examine associations between general practice population and characteristics, with the use of urgent referrals for suspected cancer, and use of endoscopy.

**Methods:**

Cross-sectional observational study of English general practices. We examined practice-level use (/1000 patients/year) of urgent referrals for suspected cancer, gastroscopy, flexible sigmoidoscopy and colonoscopy. We used mixed-effects Poisson regression to examine associations with the sociodemographic profile of practice populations and other practice attributes, including the average age, sex and country of qualification of practice doctors.

**Results:**

The sociodemographic characteristics of registered patients explained much of the between-practice variance in use of urgent referrals (32%) and endoscopic investigations (18–25%), all being higher in practices with older and more socioeconomically deprived patients. Practice-level attributes explained a substantial amount of between-practice variance in urgent referral (19%) but little of the variance in endoscopy (3%-4%). Adjusted urgent referral rates were higher in training practices and those with younger GPs. Practices with mean doctor ages of 41 and 57 years (at the 10th/90th centiles of the national distribution) would have urgent referral rates of 24.1 and 19.1/1000 registered patients, *P* < 0.001.

**Conclusion:**

Most between-practice variation in use of urgent referrals and endoscopies seems to reflect health need. Some practice characteristics, such as the mean age of GPs, are associated with appreciable variation in use of urgent referrals, though these associations do not seem strong enough to justify targeted interventions.

## Introduction

Most studies of care quality examine variations in disease management, but the importance of studying variation in the diagnostic process is increasingly recognized (1). Primary care has a pivotal role in diagnosis, as in many health care systems patients initially present to general practice (2,3). While a working diagnosis can usually be established during a consultation, investigations and referrals can form a critical part of the diagnostic process (1).

Cancer provides a useful disease model for examining the role of primary care in the diagnostic process (2,3). In England, nearly all residents are registered with a general practice, and primary care is accessed free of charge. GPs have direct access to an urgent referral pathway for patients with suspected cancer, where patients are seen by a specialist within 2 weeks of referral, and to certain specialist investigations including gastrointestinal endoscopy (4,5). There is appreciable between-practice variation in both the use of urgent referrals for suspected cancer and the use of endoscopy, beyond what can be expected by chance (4). Additionally, there is evidence for practice-level associations between higher rates of either urgent referrals for suspected cancer or gastroscopy, and improved clinical outcomes in cancer patients, including survival (5,6). Examining predictors of general practice variation in the use of referrals for suspected cancer and endoscopies is therefore important. In a recent study, we found that higher practice scores of patient-reported measures of doctor communication are associated with higher use of referrals and endoscopies in that practice, while the opposite was true for a proxy measure of care continuity (7).

In the present study, which forms part of a broader project that explores potential associations of a range of practice-based attributes with variation in measures of diagnostic activity, we examine whether the characteristics of GPs in a practice (their age, sex and country of qualification) and practice type (e.g. regarding training status and rural/urban location) are associated with the use of urgent referrals for suspected cancer or the use of endoscopic investigations, independently of the characteristics of the practice population. Therefore, our objective was to examine whether and how such variables are associated with the use of urgent referrals for suspected cancer or endoscopies, in order to acquire insights about potential mechanisms responsible for variation in diagnostic care.

## Methods

In brief, we studied all English general practices with relevant data (sample size *n* > 7000—see also Results and Table 1-footnote). We focused on four practice-level indicators, i.e. the rate (*n*/1000 registered patients/year) of urgent referrals for suspected cancer, gastroscopy, flexible sigmoidoscopy and colonosocopy. We examined whether and how the sociodemographic characteristics of registered patients and other practice-level attributes were associated with each of these four outcomes. The study year was 2013.

**Table 1.** Description of practice-level variables (diagnostic activity indicators used as outcomes variables, and practice characteristics used as exposures) used in analysis; data relate to 2013 unless otherwise noted

	Median (IQR)	10–90th centiles
Outcomes (diagnostic activity indicators)		
Urgent referrals for suspected cancer (/1000 patient-years)	20.8 (15.4–27.0)	10.7–33.1
Gastroscopy (upper gastrointestinal endoscopy) (/1000 patient-years)^a^	11.1 (8.6–14.0)	6.6–17.2
Flexible sigmoidoscopy (/1000 patient-years)^a^	4.2 (3.0–5.7)	2.0–7.6
Colonoscopy (/1000 patient-years)^a^	6.7 (5.0–8.6)	3.7–10.5
% of urgent referrals for suspected cancer that resulted in cancer diagnosis (‘conversion rate’)	*10.1 (7.6–13.2*)	*5.5–16.9*
% of treated cancer patients whose diagnosis resulted from an urgent referral for suspected cancer (‘detection rate’)	*47.4 (39.2–55.4*)	*30.8–63.0*
% of cancer patients in a practice who were diagnosed through an emergency presentation to hospital services	*23.3 (17.4–30.0*)	*11.8–37.5*
Exposures (practice population/characteristics)		
Continuous variables	Median (IQR)	10–90th centiles
List size (N of registered patients)^b^	6548 (4004–9837)	2615–12817
Number of patients per GP FTE^b^	1785 (1496–2146)	1256–2685
Mean GP age (years) calculated using mid-points of age bands^b^	47 (44–51)	41–57
% of practice GPs who are male^b^	*50 (40–67*)	*25–100*
% of practice GPs who are UK-qualified^b^	*83 (50–100*)	*0–100*
% of practice patients who are male^b^	*50 (49–51*)	*48–52*
% of practice patients who are >65 years^b^	*17 (13–21*)	*10–25*
% of practice patients who are White^c^	*96 (88–99*)	*68–99*
% of practice patients who are Mixed^c^	*0.0 (0.0–0.9*)	*0–2.0*
% of practice patients who are Asian^c^	*1.6 (0.7–5.1*)	*0–12.8*
% of practice patients who are Black^c^	*0.6 (0.0–2.0*)	*0–7.1*
% of practice patients of ‘Other’ ethnicity^c^	*0.9 (0.0–2.7*)	*0–7.2*
Categorical variables	Number (%) by variable category	
Deprivation quintile of registered practices (Q1 = least deprived, Q5 = most deprived)^d^	Q1: 1598 (22%); Q2: 1569 (22%); Q3: 1519 (21%); Q4: 1420 (20%); Q5: 1098 (15%)	
Single-handed status (Yes/No)^e^	Single-handed: 191 (3%); not single-handed: 7013 (97%)	
Practice location status (Yes/No)^f^	Urban: 5987 (83%); rural: 1217 (17%)	
Practice training status (Yes/No)^b^	Non-training practice: 5293 (74%); training practice: 1911 (27%)	

Unless otherwise noted, data relate to the 2013 public release of Cancer Services Public Health Profile, including practices with >1000 patients (see Results, 1st para). Among the 7962 practices included in the Profile, we excluded 73 with incomplete General and Personal Medical Services data, 680 had <100 GPPS respondents and 5 with missing deprivation values, resulting in a maximum analysis sample of 7204 practices. FTE, full time equivalent; HSCIC, health and social care information centre.

^a^Endoscopies carried out as day cases or inpatients (source Hospital Episodes Statistics).

^b^Based on the 2013 General and Personal Medical Services data.

^c^Using responses to the self-reported ethnicity item (question 49) of the 2012/2013 GPPS.

^d^Practice level deprivation scores for 2011 obtained from HSCIC’s indicator portal (17).

^e^Defined as practice where >50% of respondents to question 8 of the 2012/2013 GPPS indicated that ‘there is usually only one GP in my GP surgery’ (23).

^f^Binary indicator (urban/rural) obtained from HSCIC’s indicator portal; rural practices included Town & Fringe, Village and Hamlet & Isolated dwelling categories (18).

### Data

In our main analysis, we used Cancer Services Public Health Profile (2013) data on the number of urgent referrals for suspected cancer and the number of gastrointestinal endoscopies (flexible sigmoidoscopy, colonoscopy and gastroscopy) to model their practice rates (*n*/1000 registered patients/year) (Table 1) (8,9). We chose those outcomes given prior evidence for practice-level associations between the use of urgent referrals for suspected cancer, or of gastroscopy, and survival of cancer patients in a practice (5,6).

In order to examine the potential influence of the characteristics of practice population on the diagnostic activity measure of interest, we used data on the sociodemographic (age, sex, deprivation and ethnicity) profile of practice populations. In order to examine the potential influence of practice characteristics, we used data on eight practice-level attributes, comprising training status (yes/no); single-handed status (yes/no); rural/urban practice location (yes/no); mean GP age; the percentage of practice GPs who are male; the percentage of practice GPs who are UK-qualified; the number of registered patients and the number of patients per Full-Time-Equivalent GP, as a proxy measure of GP workload. For these variables, information was derived from the 2013 General and Personal Medical Services dataset, the 2012/2013 General Practice Patient Survey (GPPS), the practice index of multiple deprivation 2011 (10) and the practice rurality indicator (11) (Table 1, footnote).

### Analysis

To appreciate the relative importance of the practice population compared with practice characteristics, we first compared the between-practice variance explained by each of the two families of relevant variables. To do this, for each of the four rate indicators, we used mixed-effects Poisson regression models, initially only including a random effect for general practice. These models capture the overall underlying variation between practices after removing the role of chance due to small numbers (4). Subsequently, we compared the overall between-practice variance derived from these initial models with the between-practice variance derived from models additionally including (i) the four practice population variables examined; (ii) the eight practice characteristics variables studied and (iii) both families of variables. These comparisons allow us to estimate the proportion of the overall between-practice variance in use of urgent referrals or endoscopies that is explained by either the practice population or other practice characteristics; it underpins the interpretation of subsequent findings about associations between each of the four diagnostic activity indicators examined and the individual practice population or practice characteristics variables.

We used the final set of models (i.e. those including all studied exposure variables relating to practice population or practice team characteristics) to obtain adjusted estimates of associations. Unadjusted associations were also derived from mixed-effects Poisson models including only a single exposure variable (and random effect for practice). Because continuous exposure variables have different distributions across practices, and to facilitate comparisons of their effect sizes, we standardized their practice values, such that the rate ratios derived from the regression models correspond to 1 SD change in the exposure variable (see Supplementary Table S1).

#### Criterion of statistical and practical significance

Because our sample was large, many statistically significant associations which may be of no practical importance can be expected. There is, however, no consensus about what constitutes a practically important effect size in observational studies of variation between general practices. In the absence of such consensus, in this study, we used an effect size cut-off that was the same as in our previous practice-level analyses, focusing *a priori* on associations with rate ratio values equal or greater to a 4% difference from parity (i.e. 0.96 or smaller or 1.04 or greater) and with *P* < 0.01 (7). In using such a threshold, our aim was to focus on those findings most likely to be practically important.

#### Population impact

To help appreciate the size of associations in their natural (‘real life’) scale, we used the regression models to predict how higher/lower centile attainment of practice characteristics translates to practice differences in use of urgent referrals for suspected cancer or endoscopies. We considered hypothetical scenarios that assumed all practices in England attained the 10th and the 90th centiles of the observed distribution of the studied exposure and subsequently predicted the nationwide rates of endoscopy of urgent referrals corresponding to those centiles. We additionally illustrate the corresponding changes that could be expected for a typical English general practice with an average practice population of 8000 cases if it attained values corresponding to these (10th centile/90th centile) scenarios.

### Supplementary analysis

Additionally to the four main outcomes (i.e. rates of urgent referral for suspected cancer and rates of flexible sigmoidoscopy, colonoscopy and gastroscopy), we also considered three other diagnostic activity indicators: the proportion of urgent referrals for suspected cancer resulting in cancer diagnosis (also known as the ‘conversion rate’, a measure of liberal/conservative use of urgent referrals for suspected cancer); the proportion of all treated cancer patients in a practice which were diagnosed after an urgent referral for suspected cancer (also known as ‘detection rate’) and the proportion of cancer patients in a practice who were diagnosed through an emergency presentation to hospital services. For these three variables, our analytical approach was identical to that used for the rate variables, but, as they represent proportions, logistic models were used. Our interest in these indicators is supplementary (to support the interpretation of the findings of the main analysis) as it is not possible to change those indicators without changing the diagnostic processes that influence them.

## Results

The analysis sample comprised 7064 to 7204 practices, depending on the outcome (Table 1). There was appreciable variability across practices in all outcome and exposure variables studied (Table 1, columns 3 and 4). We hereafter consider the findings derived from the multivariable models, while univariate associations are presented in Supplementary Table S2.

### 

#### Predictors of use of urgent referrals for suspected cancer

Both population and practice characteristics explained appreciable proportions of the between-practice variance in rates of urgent referrals for suspected cancer (32% and 19%, respectively, Table 2).

**Table 2.** Change in between-practice variance in rates of urgent referrals and endoscopies, after adjustment for different groups of exposure variables; data relate to studied English general practices in 2013.

	Percentage reduction in between-practice variance		
	After adjustment for population characteristics, %	After adjustment for practice team characteristics, %	After adjustment for both population and practice/practice team characteristics, %
Rate of urgent referral for suspected cancer	31.8	19.1	40.6
Sigmoidoscopy rate	17.5	2.8	18.1
Colonoscopy rate	22.2	3.5	22.6
Gastroscopy rate	25.1	3.3	27.4

#### Population characteristics

Practices with averagely older and more deprived patients had higher rates of urgent referrals for suspected cancer [Relative Risk (RR) for a SD change in the proportion of patients aged ≥65 = 1.17; RR for deprivation quintile 5 versus 1 = 1.14; *P* < 0.001 for both, Table 3, columns 3 and 4]. Conversely, practices with higher proportions of male, Asian and ‘Other’ ethnicity patients had lower urgent referral rates (RR = 0.96, 0.96 and 0.95, respectively, *P* < 0.001 for all three).

**Table 3.** Adjusted associations between rates of urgent referrals for suspected cancer and gastrointestinal endoscopy, with practice/population characteristics, in English general practices in 2013. Coefficients for continuous variables denote a SD change in the exposure. Bold fonts used for rate ratio values ≥1.04 or ≤0.96.

	Urgent referral rate for suspected cancer— columns 2–3		Sigmoidoscopy rate— columns 4–5		Colonoscopy rate— columns 6–7		Gastroscopy rate— columns 8–9	
	RR (LCI-UCI)	*P*	RR (LCI-UCI)	*P*	RR (LCI-UCI)	*P*	RR (LCI-UCI)	*P*
Practice characteristics								
Single-handed	**0.89 (0.85–0.92**)	***<0.001***	0.95 (0.90–1.01)	*0.131*	1.02 (0.97–1.07)	*0.368*	**0.94 (0.90–0.98**)	***0.002***
Rural	1.00 (0.98–1.02)	*0.803*	**0.96 (0.93–0.98**)	***<0.001***	0.99 (0.97–1.01)	*0.425*	**0.95 (0.93–0.97**)	***<0.001***
Training	**1.04 (1.03–1.06**)	***<0.001***	**0.96 (0.94–0.98**)	***<0.001***	1.00 (0.99–1.02)	*0.695*	1.00 (0.98–1.01)	*0.491*
List size	1.01 (1.00–1.02)	*0.206*	1.01 (1.00–1.03)	*0.033*	0.99 (0.98–1.00)	*0.021*	1.00 (0.99–1.00)	*0.304*
Patients per FTE GP	1.00 (0.99–1.01)	*0.42*	1.01 (0.99–1.02)	*0.378*	0.99 (0.98–1.00)	*0.233*	0.99 (0.99–1.00)	*0.128*
Proportion male GPs	0.97 (0.96–0.98)	*<0.001*	1.01 (0.99–1.02)	*0.234*	0.99 (0.98–1.00)	*0.039*	0.99 (0.98–0.99)	*0.001*
Proportion of GPs trained in UK	1.03 (1.02–1.04)	*<0.001*	1.01 (1.00–1.03)	*0.0369*	1.00 (0.99–1.01)	*0.8707*	1.01 (1.00–1.02)	*0.0344*
Mean GP age	**0.91 (0.90–0.92**)	***<0.001***	0.98 (0.96–0.99)	*0.002*	0.99 (0.97–1.00)	*0.008*	**0.96 (0.95–0.97**)	***<0.001***
Population characteristics								
Male %	**0.96 (0.95–0.97**)	***<0.001***	1.01 (0.99–1.02)	*0.439*	**0.96 (0.95–0.97**)	***<0.001***	0.99 (0.98–1.00)	*0.197*
Aged 65 or older %	**1.17 (1.16–1.18**)	***<0.001***	**1.15 (1.14–1.17**)	***<0.001***	**1.14 (1.13–1.16**)	***<0.001***	**1.16 (1.15–1.18**)	***<0.001***
Mixed %	1.00 (0.99–1.01)	*0.498*	0.98 (0.97–0.99)	*0.007*	1.00 (0.99–1.01)	*0.443*	0.98 (0.97–0.99)	*<0.001*
Asian %	**0.96 (0.95–0.97**)	***<0.001***	0.99 (0.97–1.01)	*0.252*	0.97 (0.96–0.98)	*<0.001*	0.99 (0.98–1.00)	*0.142*
Black %	0.99 (0.98–1.00)	*0.15*	0.97 (0.96–0.99)	*0.002*	0.98 (0.97–0.99)	*0.002*	0.97 (0.96–0.98)	*<0.001*
Other %	**0.95 (0.94–0.96**)	***<0.001***	**0.94 (0.92–0.96**)	***<0.001***	0.98 (0.96–0.99)	*0.003*	0.98 (0.97–1.00)	*0.014*
Deprivation Quintile 2	**1.05 (1.03–1.06**)	***<0.001***	**1.05 (1.03–1.08**)	***<0.001***	1.02 (1.00–1.04)	*0.052*	**1.05 (1.03–1.07**)	***<0.001***
Deprivation Quintile 3	**1.09 (1.06–1.11**)	***<0.001***	**1.04 (1.01–1.07**)	***0.002***	**1.06 (1.04–1.08**)	***<0.001***	**1.13 (1.11–1.15**)	***<0.001***
Deprivation Quintile 4	**1.11 (1.08–1.13**)	***<0.001***	**1.10 (1.07–1.13**)	***<0.001***	**1.12 (1.09–1.14**)	***<0.001***	**1.23 (1.20–1.25**)	***<0.001***
Deprivation Quintile 5	**1.14 (1.11–1.17**)	***<0.001***	**1.11 (1.07–1.14**)	***<0.001***	**1.08 (1.06–1.11**)	***<0.001***	**1.29 (1.26–1.32**)	***<0.001***

FTE, full time equivalent; LCI, lower confidence interval; UCI, upper confidence interval.

#### Practice characteristics

Practices with older doctors had lower urgent referral rates (RR for a SD change in mean practice GP age = 0.91, *P* < 0.001). Training practices had higher rates of urgent referrals (RR compared with non-training practices = 1.04, *P* < 0.001), whereas single-handed practices had lower rates compared with non-single-handed practices (RR = 0.89, *P* < 0.001). There was evidence (*P* < 0.001) for associations with the percentage of male GPs and the percentage of UK-qualified GPs, but effect sizes were smaller than our criterion of practical significance (RR = 0.97 and 1.03, respectively). There was no evidence of associations with practice rural/urban location, list size or the number of patients per full-time equivalent GP.

#### Predictors of gastrointestinal endoscopy use

While population characteristic variables explained an appreciable proportion of between-practice variance in use of all three endoscopic investigations considered (18% to 25%), practice characteristics explained much smaller proportions (3% to 4%, Table 2).

#### Population characteristics

Practices with more deprived patients had higher rates of endoscopy use (RR for practices in the most versus the least deprived quintiles: 1.11 for flexible sigmoidoscopy, 1.08 for colonoscopy and 1.29 for gastroscopy, *P* < 0.001 for all, Table 3, columns 4–9). Practices with older patients also had higher endoscopy rates (RR for a SD change in mean practice GP age = 1.15 for flexible sigmoidoscopy, 1.14 for colonoscopy and 1.16 for gastroscopy, *P* < 0.001 for all three).

#### Practice characteristics

There was statistical evidence of appreciable associations for only 5 of 24 possible associations (8 practice characteristics × 3 endoscopy indicators), without consistent patterns across the three types of gastrointestinal endoscopy.

### Population impact illustration

The observed differences translate to small absolute differences in referral rates but appreciable relative differences between practices (Table 5). For example, after adjusting for all other variables, and assuming a causal effect, practices with mean doctor ages of 41 and 57 years (i.e. at the 10th/90th centiles of the national distribution of mean GP age in a practice) would be expected to have urgent referral rates of 24.1 and 19.1 per 1000 registered patients. This represents a small absolute difference (of 5 referrals/1000 registered patients/year) but an appreciable relative difference (around 20%) in urgent referral activity.

### Supplementary analyses regarding outcomes of the diagnostic process

#### ‘Conversion’ and ‘detection’ rates

Practice team characteristics associated with higher rates of urgent referral for suspected cancer were generally also associated with lower proportions of urgently referred patients, who were found to have cancer (lower ‘conversion rates’), and higher proportions of all cancer patients detected after urgent referral (higher ‘detection rates’, Table 4, columns 2–5). In contrast, for practice population characteristics, variables associated with higher rates of urgent referrals for suspected cancer were not consistently associated with neither conversion nor detection rates. For example, though increasing deprivation of the practice population was associated with higher rates of urgent referrals for suspected cancer, no associations were apparent for either ‘conversion’ or ‘detection’ rates.

**Table 4.** Adjusted associations between secondary outcomes, with practice/population characteristics, in English general practices in 2013. Coefficients for continuous variables denote a SD change in the exposure variable. Bold fonts used for rate ratio values ≥1.04 or ≤0.96

	Proportion of urgently referred patients in a practice who were diagnosed with cancer (‘conversion rate’)—columns 10–11		Proportion of all cancer patients in a practice diagnosed after an urgent referral (‘detection rate’)—columns 12–13		Proportion of cancer patients in a practice diagnosed after an emergency presentation—columns 14–15	
	RR (LCI-UCI)	*P*	RR (LCI-UCI)	*P*	OR (LCI-UCI)	*P*
Practice characteristics						
Single-handed	1.05 (0.99–1.13)	*0.127*	**0.89 (0.82–0.96**)	***0.002***	1.02 (0.94–1.10)	*0.688*
Rural	1.00 (0.98–1.02)	*0.768*	1.02 (1.00–1.05)	*0.035*	**0.96 (0.94–0.98**)	***0.001***
Training	**0.96 (0.95–0.98**)	***<0.001***	1.00 (0.98–1.02)	*0.793*	0.98 (0.96–1.00)	*0.048*
List size	1.01 (1.00–1.02)	*0.182*	1.01 (1.00–1.02)	*0.116*	0.99 (0.98–1.00)	*0.015*
Patients per FTE GP	1.00 (0.99–1.01)	*0.859*	1.00 (0.99–1.01)	*0.8*	0.99 (0.98–1.00)	*0.123*
Proportion male GPs	1.02 (1.01–1.03)	*0.003*	0.98 (0.96–0.99)	*0.001*	1.02 (1.00–1.03)	*0.031*
Proportion of GPs trained in UK	0.99 (0.98–1.00)	*0.0794*	**1.04 (1.02–1.05**)	***<0.001***	0.97 (0.95–0.98)	*<0.001*
Mean GP age	**1.06 (1.05–1.08**)	***<0.001***	**0.95 (0.94–0.96**)	***<0.001***	0.99 (0.98–1.01)	*0.388*
Population characteristics						
Male %	**1.05 (1.04–1.07**)	***<0.001***	1.00 (0.98–1.01)	*0.504*	1.01 (1.00–1.03)	*0.088*
Aged 65 or older %	**1.12 (1.11–1.14**)	***<0.001***	1.02 (1.01–1.04)	*0.008*	0.98 (0.97–1.00)	*0.044*
Mixed %	0.97 (0.96–0.98)	*<0.001*	0.99 (0.98–1.01)	*0.35*	0.98 (0.97–1.00)	*0.013*
Asian %	**0.96 (0.95–0.98**)	***<0.001***	0.99 (0.97–1.01)	*0.354*	1.00 (0.98–1.03)	*0.694*
Black %	1.00 (0.99–1.02)	*0.825*	1.03 (1.01–1.05)	*0.009*	1.01 (0.99–1.03)	*0.182*
Other %	0.97 (0.95–0.99)	*0.002*	0.97 (0.95–1.00)	*0.028*	0.99 (0.96–1.01)	*0.378*
Deprivation Quintile 2	1.01 (0.99–1.03)	*0.217*	1.01 (0.99–1.04)	*0.205*	**1.04 (1.02–1.07**)	***0.001***
Deprivation Quintile 3	1.02 (1.00–1.04)	*0.132*	**1.04 (1.01–1.06**)	***0.003***	**1.10 (1.08–1.13**)	***<0.001***
Deprivation Quintile 4	1.02 (0.99–1.04)	*0.178*	1.02 (1.00–1.05)	*0.079*	**1.20 (1.17–1.23**)	***<0.001***
Deprivation Quintile 5	1.01 (0.99–1.04)	*0.345*	1.02 (0.99–1.06)	*0.138*	**1.29 (1.25–1.33**)	***<0.001***

FTE, full time equivalent; LCI, lower confidence interval; UCI, upper confidence interval.

#### Diagnosis of cancer through emergency presentation

The practice’s deprivation quintile was strongly associated with the proportion of patients diagnosed through emergency presentation (deprivation quintile 5 versus quintile 1 odds ratio = 1.29; *P* < 0.001, Table 4, columns 6 and 7). Rural practices had lower proportions of patients diagnosed through emergency presentation (RR = 0.96, *P* < 0.001), without appreciable associations for any of the other (seven) practice characteristics studied, including the proportion of registered patients who belong to ethnic minorities.

## Conclusions

### Summary of main findings

Practice population characteristics explain much of the variation in the studied outcomes, with general practices serving older and more deprived patient populations tending to have higher use of both urgent referrals for suspected cancer and endoscopies. Practice characteristics, on the other hand, explain appreciable variation in use of urgent referrals for suspected cancer, but not endoscopy use. Practices with younger GPs and training practices generally had higher rates of urgent referrals for suspected cancer, whereas the opposite was true for single-handed practices. No appreciable associations were apparent between use of urgent referrals for suspected cancer and the other five practice characteristics studied (urban/rural practice location, list size and GP workload, percentage of male GPs in a practice and percentage of UK-qualified GPs), though there was statistical evidence for weak associations for the latter two.

### Comparison with previous research findings

Three recent US studies have examined the influence of doctors’ age, sex and country of qualification on patient outcomes, but the setting of care (i.e. hospital), methodology and outcome measures were markedly different; therefore, they are only tangentially relevant to our study (12–14). Higher practice-level proportions of female GPs were associated with longer diagnostic intervals in Denmark and a higher probability of advanced stage at diagnosis in England (15,16). Our findings, however, suggest that female and male GPs do not vary substantially in decisions about urgent referrals or endoscopic investigations. Regarding the rate of urgent referrals for suspected cancer, a recent study examining differences between training and non-training practices in England, similarly to our study, found higher use of in training practices (17). A higher burden of emergency hospital admissions relating to the diagnosis of cancer between 2007 and 2009 was reported in practices with higher proportions of non-UK qualified GPs (18), but in our study and examining more recent (2013) data, we found only weak associations that are unlikely to be of practical significance. Higher levels of general emergency hospital admissions (not necessarily in patients subsequently diagnosed with cancer) are associated with higher levels of deprivation of practice populations and greater general practice proximity to hospital (a proxy indicator for non-rural location) (19–21). The fact that practice team characteristics associated with higher rates of urgent referral for suspected cancer were generally also associated with lower proportions of urgently referred patients who were found to have cancer and higher proportions of all cancer patients detected after urgent referral is concordant with prior-related evidence also reporting similar practice-level associations between these metrics (5,9). This is indeed expected if one considers an urgent referral to represent a diagnostic test: if the true disease risk remains constant, greater use of the test will result in a lower positive predictive value (PPV)—known in this context as a ‘conversion rate’ (i.e. fewer investigated patients being found to have cancer) and greater sensitivity (greater number of cases detected through urgent referrals) and vice versa.

### Strengths and limitations

The strengths of our study include the use of objectively defined outcomes and practice population and team characteristics in a large nationwide sample of practices. We had no access to patient-level data, so we could not adjust for the characteristics of patients that were investigated or referred at the individual level, although we used practice-level measures of age, gender and ethnic and deprivation groups. The studied associations may differ in the small number of practices that were not included in our analyses, though this does not undermine the validity of the findings regarding the great majority of English practices that were included. Ecological studies are often under-powered, but our study includes a large number of practices, increasing the power to detect associations if those are present (22). By the nature of our study, we were not able to examine the appropriateness of the diagnostic management in referred/non-referred or investigated/non-investigated patients. We used a cut-off value for associations that are likely to be of practical importance, beyond statistical significance (7). Using such thresholds is often necessary (and indeed recommended) in studies using large samples examining multiple exposures. Lastly, while we used 1 year of data, future studies may examine potential changes in the observed associations over time, particularly before/after the implementation of national referral guidelines for suspected cancer issued in 2015 (23).

### Interpretation and implications

It is important to appreciate that, because both urgent referrals and endoscopies are relatively infrequent, the size of the associations we describe is small in terms of absolute differences but substantive in relative terms (see Table 5, columns 4 and 5). This means that, for example, the doctor’s age may only have a small influence on the diagnostic process of the average consultee, but a sizeable relative impact across the population and the health care system, particularly when considering the resource impact associated with 20% variation in demand.

**Table 5.** Expected indicator values if all practices changed category (binary variables) or moved from the 10th to the 90th percentile, of the distribution of the practice characteristic of interest, and illustrations of effects for a typical English practice serving 8000 patients during 2013

	10th percentile/No*	90th percentile/Yes*	Absolute difference in rate/ Yes-No*—column 4	Relative difference in rate (%)—column 5	Absolute difference for a typical and averagely sized practice (of 8,000 patients) hypothetically moving from the 10^th^ centile the 90^th^ centile
Sigmoidoscopy rate (*N*/1000 patient-years)					
Rural	*4.7*	*4.4*	*−0.3*	*−6.0*	*−2.2*
Training	*4.7*	*4.5*	*−0.2*	*−5.3*	*−2.0*
Gastroscopy rate (*N*/1000 patient-years)					
Single-handed	*11.6*	*10.6*	*−1.0*	*−8.7*	*−8.0*
Rural	*11.7*	*10.9*	*−0.8*	*−6.7*	*−6.2*
Mean GP age	12.0	10.8	*−*1.2	*−*9.7	*−9.3*
Urgent referral rate for suspected cancer (*n*/1000 patient-years)					
Single-handed	*22.3*	*18.9*	*−3.4*	*−15.1*	*−27.0*
Training	*21.8*	*23.1*	*+1.3*	*+5.9*	*10.2*
Mean GP age	24.1	19.1	*−*5.0	*−*20.8	*−40.2*

Reported values are adjusted for exposure variables (Table 1) and are on the relevant scale for each indicator, i.e. either rate [(*n*/1000 registered patients) or percentage]. Only variables with effect sizes ≥1.04 or ≤0.96/change visualized.

*For binary characteristics, the absence and presence of the characteristic are shown.

The fact that practices with older and more deprived patients tend to have higher use of endoscopies and urgent referrals are likely to reflect variation appropriate to the level of health care needs, particularly as cancer incidence increases with both age and deprivation (not least for smoking-related cancers). It appears that decision-making by GPs about the use of urgent referrals and endoscopies is generally well-calibrated to the sociodemographic profile of their patients. This is evident given that although practices serving more deprived populations tend to refer patients urgently for suspected cancer at greater rate than practices serving relatively affluent patients, this is not associated with lower proportion of cancer diagnosis among referred patients (Tables 3 and 4). This means that the increase in urgent referral activity is proportionate to increase in risk, leaving the percentage of tested patients who are found to have cancer unchanged. This also means that doctors in practices with patients who are older than average and live in areas that are more deprived than average tend to have appropriately higher rate of use of urgent referrals, proportionate to greater need (i.e. higher cancer risk). The observed lower rates of urgent referrals for suspected cancer and endoscopies in practices with higher proportion of ethnic minorities may indicate a degree of unmet need.

Although distance to hospital may limit the use of hospital services in rural populations, we did not observe differences in the rate of urgent referrals for suspected cancer between rural and urban practices. Training practices had higher use of urgent referral rates for suspected cancer, which may reflect greater degree of compliance of referral guidelines for suspected cancer, or their more avid interpretation. Differences in urgent referral rates for suspected cancer between practices whose doctors vary by age may reflect that GPs of different seniority have different levels of professional experience or their era of medical training. The findings suggest that older doctors may be more comfortable in dealing with uncertainty in the diagnostic process (24), as they are more likely to opt for expectant management without recourse to specialist investigation or referral. Given that older doctors tend to have lower than average referral rates, the findings would suggest that educational interventions aimed at younger doctors (or, even, medical students) are unlikely to address variation in referral rates.

The optimal level of use of urgent referrals and endoscopies cannot be determined by our study, although it reveals likely mechanisms responsible for related variation. Nonetheless, the 2015 NICE guidelines for suspected cancer in primary care implicitly suggest that in England, greater use of urgent referrals than in the past would be desirable (23).

We conclude that much of between-practice variation in use of urgent referrals and endoscopies reflects sociodemographic variation in patient populations. Interventions aimed at increasing the rates of referrals for suspected cancer in low referring practices seem justified given prior evidence indicating associations with clinical outcomes in cancer patients. However, only a few practice characteristics, such as the mean age of GPs, are associated with appreciable variation in use of urgent referrals. The number and strength of such associations seem inadequate to justify targeting of practice-level interventions.

## Supplementary Material

cmy118_suppl_Supplementary_data_table

## Declaration

Funding: the research was supported by or relates to the following grants or awards: Cancer Research UK/National Awareness and Early Diagnosis Initiative Project (grant no. C18081/A17854); Cancer Research UK Advanced Clinician Scientist Fellowship (no. C18081/A18180); Cancer Research UK Population Research Catalyst Award (*CanTest* Research Collaborative) C8640/A23385; Houston VA HSR&D Center for Innovations in Quality, Effectiveness and Safety CIN 13–413. The funders had no influence on study design, analysis and interpretation of findings.

Ethical approval: ethics approval was not required for this study.
