# Supplementary material for: Associations between general practice characteristics with use of urgent referrals for suspected cancer and endoscopies: a cross-sectional ecological study
Source: Fam Pract. 2018 Dec 12;36(5):573–80. doi: 10.1093/fampra/cmy118 (PMC6781939; doi:10.1093/fampra/cmy118)
Supplement: cmy118_suppl_Supplementary_data_table [file cmy118_suppl_supplementary_data_table.pdf]

Supplementary Table S1

Standardisation of exposure variables to enable comparability of effect sizes

For continuous exposure variables each have a different distribution across practices, the effect sizes of the respective associations are not directly comparable. Therefore, we standardised (unshrunk) practice values for these variables in the regression models. We standardised by first centring at the mean of the distribution (i.e. for each practice, we subtracted the mean value across all practices from its actual value), and then dividing by 1.35 standard deviations. An one unit difference in the resulting standardised scores corresponds to a change between the 25th and 75th percentile of normally distributed continuous variables. When using these standardised scores in regression, for either rate or proportion indicators, the resulting rate or odds ratios correspond to the change (in the rate or the odds) resulting from moving from the 25th to the 75th centile of the exposure variable (practice team or practice population characteristic) of interest, if it is normally distributed.

Supplementary Table S2: Unadjusted associations between diagnostic activity indicators and practice team and practice's population characteristics. The coefficients for numerical variables reflect a Standard Deviation change in the exposure.

	**Sigmoidoscopy rate**		**Colonoscopy rate**		**Gastroscopy rate**		**Urgent referral rate for suspected cancer**		**Proportion of urgently referred patients in a practice who were diagnosed with cancer ('conversion rate')**		**Proportion of all cancer patients in a practice diagnosed after an urgent referral ('detection rate')**		**Proportion of cancer patients in a practice diagnosed after an emergency presentation**	
	**RR (LCI-UCI)**	**P**	**RR (LCI-UCI)**	**P**	**RR (LCI-UCI)**	**P**	**RR (LCI-UCI)**	**P**	**RR (LCI-UCI)**	**P**	**RR (LCI-UCI)**	**P**	**OR (LCI-UCI)**	**P**
Practice characteristics														
Single handed	0.96 (0.91-1.03)	0.260	1.00 (0.95-1.05)	0.953	**0.91 (0.87-0.96)**	**<0.001**	**0.73 (0.70-0.77)**	**<0.001**	**1.25 (1.17-1.34)**	**<0.001**	**0.78 (0.73-0.84)**	**<0.001**	**1.12 (1.03-1.21)**	**0.006**
Rural	**1.08 (1.06-1.11)**	**<0.001**	**1.10 (1.08-1.12)**	**<0.001**	1.02 (1.01-1.04)	0.009	**1.17 (1.14-1.19)**	**<0.001**	**1.11 (1.09-1.13)**	**<0.001**	**1.05 (1.03-1.07)**	**<0.001**	**0.88 (0.86-0.90)**	**<0.001**
Training	0.98 (0.97-1.00)	0.109	1.02 (1.00-1.03)	0.025	1.01 (0.99-1.02)	0.252	**1.13 (1.11-1.15)**	**<0.001**	**0.93 (0.92-0.95)**	**<0.001**	**1.04 (1.02-1.06)**	**<0.001**	**0.94 (0.93-0.96)**	**<0.001**
List size	1.03 (1.01-1.04)	<0.001	1.01 (1.00-1.02)	0.042	1.01 (1.00-1.02)	0.009	**1.09 (1.08-1.10)**	**<0.001**	0.98 (0.97-0.99)	<0.001	1.03 (1.02-1.04)	<0.001	**0.96 (0.95-0.97)**	**<0.001**
Patients per FTE GP	0.97 (0.96-0.98)	<0.001	**0.96 (0.95-0.97)**	**<0.001**	**0.96 (0.95-0.97)**	**<0.001**	**0.92 (0.91-0.93)**	**<0.001**	1.00 (0.98-1.01)	0.480	0.97 (0.95-0.98)	<0.001	1.02 (1.01-1.03)	0.006
Proportion male GPs	1.01 (1.00-1.03)	0.031	0.99 (0.98-1.00)	0.036	0.99 (0.98-1.00)	0.067	**0.92 (0.91-0.93)**	**<0.001**	**1.07 (1.06-1.08)**	**<0.001**	**0.95 (0.94-0.96)**	**<0.001**	**1.05 (1.04-1.07)**	**<0.001**
Proportion of GPs trained in UK	**1.06 (1.05-1.07)**	**<0.001**	**1.05 (1.04-1.06)**	**<0.001**	**1.04 (1.03-1.05)**	**<0.001**	**1.16 (1.14-1.17)**	**<0.001**	0.99 (0.98-1.00)	0.030	**1.08 (1.06-1.09)**	**<0.001**	**0.91 (0.90-0.92)**	**<0.001**
Mean GP age	**0.96 (0.95-0.97)**	**<0.001**	0.97 (0.96-0.98)	<0.001	**0.95 (0.94-0.96)**	**<0.001**	**0.85 (0.84-0.86)**	**<0.001**	**1.08 (1.07-1.09)**	**<0.001**	**0.92 (0.91-0.93)**	**<0.001**	**1.04 (1.03-1.06)**	**<0.001**
Practice population characteristics														
Male	**0.96 (0.95-0.97)**	**<0.001**	**0.93 (0.92-0.94)**	**<0.001**	0.97 (0.96-0.97)	<0.001	**0.87 (0.86-0.88)**	**<0.001**	1.03 (1.02-1.05)	<0.001	**0.95 (0.94-0.97)**	**<0.001**	**1.07 (1.06-1.09)**	**<0.001**
Aged 65 or older	**1.19 (1.17-1.20)**	**<0.001**	**1.17 (1.16-1.18)**	**<0.001**	**1.14 (1.13-1.15)**	**<0.001**	**1.22 (1.21-1.23)**	**<0.001**	**1.15 (1.14-1.17)**	**<0.001**	**1.04 (1.02-1.05)**	**<0.001**	**0.93 (0.92-0.94)**	**<0.001**
Mixed	**0.96 (0.95-0.97)**	**<0.001**	0.98 (0.97-0.99)	<0.001	**0.96 (0.95-0.97)**	**<0.001**	0.98 (0.97-0.99)	<0.001	0.95 (0.93-0.96)	<0.001	0.99 (0.98-1.01)	0.269	0.98 (0.97-1.00)	0.041
Asian	0.97 (0.95-0.98)	<0.001	**0.94 (0.93-0.95)**	**<0.001**	**0.96 (0.95-0.97)**	**<0.001**	**0.91 (0.90-0.92)**	**<0.001**	**0.96 (0.94-0.98)**	**<0.001**	0.97 (0.95-0.99)	0.006	1.01 (0.99-1.04)	0.202
Black	**0.95 (0.94-0.97)**	**<0.001**	0.97 (0.96-0.98)	<0.001	0.97 (0.96-0.98)	<0.001	0.97 (0.96-0.98)	<0.001	0.98 (0.96-0.99)	0.003	1.01 (1.00-1.03)	0.113	**1.05 (1.03-1.08)**	**<0.001**
Other	**0.92 (0.90-0.94)**	**<0.001**	**0.95 (0.93-0.96)**	**<0.001**	0.97 (0.95-0.98)	<0.001	**0.91 (0.90-0.93)**	**<0.001**	**0.95 (0.93-0.97)**	**<0.001**	**0.96 (0.94-0.99)**	**0.003**	1.03 (1.00-1.06)	0.046
Quintile 2	**1.04 (1.02-1.07)**	**0.002**	1.01 (0.99-1.03)	0.469	1.04 (1.02-1.06)	<0.001	1.02 (1.00-1.04)	0.112	1.01 (0.99-1.03)	0.314	1.00 (0.98-1.02)	0.872	**1.05 (1.03-1.08)**	**<0.001**
Quintile 3	0.98 (0.95-1.00)	0.094	0.99 (0.97-1.01)	0.334	**1.06 (1.04-1.08)**	**<0.001**	0.97 (0.95-0.99)	0.003	0.99 (0.97-1.01)	0.260	1.00 (0.98-1.02)	0.973	**1.14 (1.11-1.16)**	**<0.001**
Quintile 4	0.97 (0.95-1.00)	0.045	0.99 (0.97-1.01)	0.303	**1.09 (1.06-1.11)**	**<0.001**	**0.91 (0.89-0.93)**	**<0.001**	**0.95 (0.93-0.98)**	**<0.001**	0.97 (0.95-0.99)	0.009	**1.25 (1.22-1.28)**	**<0.001**
Quintile 5	**0.91 (0.89-0.94)**	**<0.001**	**0.90 (0.88-0.92)**	**<0.001**	**1.07 (1.04-1.09)**	**<0.001**	**0.86 (0.84-0.88)**	**<0.001**	**0.92 (0.89-0.94)**	**<0.001**	**0.95 (0.92-0.98)**	**<0.001**	**1.37 (1.33-1.41)**	**<0.001**
